# Prebiotic Effects of Wheat Arabinoxylan Related to the Increase in Bifidobacteria, Roseburia and Bacteroides/Prevotella in Diet-Induced Obese Mice

**DOI:** 10.1371/journal.pone.0020944

**Published:** 2011-06-09

**Authors:** Audrey M. Neyrinck, Sam Possemiers, Céline Druart, Tom Van de Wiele, Fabienne De Backer, Patrice D. Cani, Yvan Larondelle, Nathalie M. Delzenne

**Affiliations:** 1 Metabolism and Nutrition Research Group, Louvain Drug Research Institute, Université catholique de Louvain, Brussels, Belgium; 2 Laboratory of Microbial Ecology and Technology, Ghent University, Ghent, Belgium; 3 Institut des Sciences de la Vie, Université catholique de Louvain, Louvain-la-Neuve, Belgium; University College Dublin, Ireland

## Abstract

**Background:**

Alterations in the composition of gut microbiota - known as dysbiosis - has been proposed to contribute to the development of obesity, thereby supporting the potential interest of nutrients targeting the gut with beneficial effect for host adiposity. We test the ability of a specific concentrate of water-extractable high molecular weight arabinoxylans (AX) from wheat to modulate both the gut microbiota and lipid metabolism in high-fat (HF) diet-induced obese mice.

**Methodology/Principal Findings:**

Mice were fed either a control diet (CT) or a HF diet, or a HF diet supplemented with AX (10% w/w) during 4 weeks. AX supplementation restored the number of bacteria that were decreased upon HF feeding, i.e. *Bacteroides-Prevotella* spp. and *Roseburia* spp. Importantly, AX treatment markedly increased caecal bifidobacteria content, in particular *Bifidobacterium animalis lactis*. This effect was accompanied by improvement of gut barrier function and by a lower circulating inflammatory marker. Interestingly, rumenic acid (C18:2 c9,t11) was increased in white adipose tissue due to AX treatment, suggesting the influence of gut bacterial metabolism on host tissue. In parallel, AX treatment decreased adipocyte size and HF diet-induced expression of genes mediating differentiation, fatty acid uptake, fatty acid oxidation and inflammation, and decreased a key lipogenic enzyme activity in the subcutaneous adipose tissue. Furthermore, AX treatment significantly decreased HF-induced adiposity, body weight gain, serum and hepatic cholesterol accumulation and insulin resistance. Correlation analysis reveals that *Roseburia* spp. and *Bacteroides/Prevotella* levels inversely correlate with these host metabolic parameters.

**Conclusions/Significance:**

Supplementation of a concentrate of water-extractable high molecular weight AX in the diet counteracted HF-induced gut dysbiosis together with an improvement of obesity and lipid-lowering effects. We postulate that hypocholesterolemic, anti-inflammatory and anti-obesity effects are related to changes in gut microbiota. These data support a role for wheat AX as interesting nutrients with prebiotic properties related to obesity prevention.

## Introduction

Recent studies demonstrated that diet-induced obesity was linked to changes in the gut microbial ecology, resulting in an increased capacity of the distal gut microbiota to promote host adiposity [1], [2]. We have previously shown that inulin-type fructans, non-digestible carbohydrates obtained from chicory root, restore the drop of bifidobacteria numbers occurring in the caeco-colon of high fat/carbohydrate-free diet-fed mice and thereby improves metabolic alterations associated with obesity, including dyslipidemia, impaired gut permeability, endotoxemia, inflammation and diabetes [3]–[6]. Inulin-type fructans are typically studied as they were the first compounds to respond to the prebiotic concept, defined as the selective stimulation of growth and/or activity of one or a limited number of microbial genus(era)/species in the gut microbiota that confer(s) health benefits to the host [7]. Other non-digestible/fermented carbohydrates, which are gradually fermented throughout the colon and which can be applied in different food matrices, may be valuable alternative substrates to test for their health effects related to their influence on gut microbiota composition.

AX are the most important non-digestible carbohydrates present in wheat. They represent 50% of dietary fibers and are mostly present in the bran and aleurone fractions [8]. AX are selectively degraded in the colon by intestinal bacteria possessing AX-degrading enzymes such as xylanases and arabinofuranosidases and represent a new class of potential prebiotics [9]–[11]. Existing in different forms, ranging from soluble to insoluble fibers and high-molecular weight to enzymatically modified short-chain fractions, the physiological effects of AX are largely unknown [12]. However, several studies indicate that they behave like fermentable fibers in the colon, with different fermentation profiles depending on the physicochemical properties and degree of polymerization, and with potential impact on lipid and glucose metabolism [8], [9], [13]–[16]. Molecular weight has been shown to play an important role, with highest activity for high-molecular weight AX [17]. Accordingly, the purpose of this study was to examine the effects of an AX concentrate, containing long-chain water-extractable AX, on the gut microbiota and lipid metabolism with focus on the expression of genes relevant to energy homeostasis and fat storage in a mice model of HF diet-induced obesity. We have chosen high molecular weight AX concentrate because previous in vitro studies have described an efficient fermentation of the AX, leading to stimulation of specific stimulation of certain bifidobacterial species and specific fermentation profiles (high propionate production) using Simulator of Human Intestinal Microbial Ecosystem (SHIME) [18], [19].

## Materials and Methods

### Ethics Statement

The animal experiments were approved by the local ethic committee; the agreement of the animal experiments performed in this study was given by the ethical committee for animal care of the Health Sector of the Université catholique de Louvain, under the supervision of prof. P. Gianello P et JP Dehoux under the specific number ULC/MD/2007/003. Housing conditions were as specified by the Belgian Law of 14 November, 1993 on the protection of laboratory animals (agreement n° LA 1230314).

### Animals and diet intervention

Twenty four male C57bl6/J mice (9 weeks old at the beginning of the experiment, Charles River Laboratories, France) were housed in groups of 4 per cage in a controlled environment (12-hour daylight cycle) with free access to food and water. After one week of acclimatisation, the mice were divided into 3 groups (n = 8/group): a control group (CT), fed with a control diet (AO4, SAFE, Villemoison-sur-Orge, France), a group fed a HF diet and a group fed the same HF diet, supplemented with AX (90% HF (w/w)+10%AX; HF-AX group). The full composition of both the HF diet (D12492, Research Diets) and the A04 standard diet was given in the Table S1. The energy content of the HF diet consisted of fat for 60%, carbohydrate for 20% and protein for 20%. BioActor (Ghent, Belgium) supplied AX (Naxus®, batch 07NX-001) with a purity of 62%, degree of substitution of 0.7, and a varying degree of polymerization, with an average above 60; the composition of the batch used for the study was 67% non-starch polysaccharides (62% AX), 18% protein, 0.5% lipids, 3.8% ash. Food intake was recorded taking into account spillage twice a week during 4 weeks. The total caloric intake was obtained by multiplying total food intake (g) for 4 mice per cage (n = 2) by the caloric value of the diets, i.e. 3.10 Kcal/g, 5.24 Kcal/g and 4.83 Kcal/g for CT, HF and HF-AX, respectively. The caloric value of the HF-AX diet was calculated taking in to account that this diet was composed of 90% HF diet and 10% AX. After 4 weeks and a 6-hour period of fasting, mice were anesthetised (ketamine/xylazine i.p., 100 and 10 mg/kg, respectively) and blood samples were harvested for further analysis. Liver, three types of white adipose tissues (visceral, epididymal and subcutaneous), *vastus lateralis* muscle, jejunum and caecum were carefully dissected and weighted before immersion in liquid nitrogen before storage at −80°C.

### Oral glucose tolerance test (OGTT)

After 3 weeks of treatment, an oral glucose tolerance test was performed on 6 h fasted-mice. Glucose was administered orally (3 g/kg body weight, 66% glucose solution) and blood glucose levels were determined using a glucose meter (Roche Diagnostics) on 3.5 µl of blood collected from the tip of the tail vein both before (−30 min and 0 min) and after glucose administration (15, 30, 60, 90, 120 min). Twenty microliters of blood were sampled 30 min before and 15 min after the glucose load to assess plasma insulin concentrations.

### Microbial analysis of the caecal contents

At the end of the experiment, the total caecum content was collected and weighed before storage at −80°C. For analysis of the microbial content, metagenomic DNA was extracted from the caecal content of all mice, using the QIAamp DNA stool mini kit (Qiagen, Venlo, The Netherlands) according to the manufacturer's instructions. Denaturing Gradient Gel Electrophoresis (DGGE) on total bacteria, bifidobacteria, lactobacilli and *Bacteroides-Prevotella* spp. was performed to study the qualitative effect of the treatment on the structure and composition of the intestinal microbial community [20]. DGGE with a 45–60% denaturing gradient was used to separate the polymerase chain reaction (PCR) products obtained with a nested approach for the 16S rRNA genes of bifidobacteria (primers BIF164f-BIF662r), lactobacilli (SGLAB0159f-SGLAB0667r) and the *Bacteroides-Prevotella* cluster (FD1-RBacPre) [21]. The first PCR round was followed by a second amplification with primers 338F-GC and 518R. The latter primers were also used to amplify the 16S rDNA of all bacteria on total extracted DNA. The DGGE patterns obtained were subsequently analyzed using the Bionumerics software version 5.10 (Applied Maths, Sint-Martens-Latem, Belgium). In brief, the calculation of the similarities was based on the Pearson (product–moment) correlation coefficient. Clustering analysis was performed using the unweighted pair group method with arithmetic mean clustering algorithm (UPGMA) to calculate the dendrograms of each DGGE gel and a combination of all gels. The latter was performed on a created composite dataset. The composite dataset of the 3 DGGE patterns was also used to perform principal component analysis (PCA). PCA ordinations were calculated using the Pearson product-moment correlation coefficient. Within each character set, this coefficient subtracts each character from the average value, and divides it by the variance of the character set.

Quantitative PCR (Q-PCR) was performed to study the quantitative effect of the treatment on the composition of the intestinal microbial community. The Q-PCR for total bacteria (using primers PRBA338f and P518r) and specific for bifidobacteria were performed as reported by Possemiers et al. [22]. The Q-PCR for *Roseburia* spp. was performed as described before [23], using the primers Ros-F1 and Ros-R1, and the Power SYBR Green PCR Master kit (Applied Biosystems, Foster City). The Q-PCR for *Bacteroides-Prevotella* spp. was performed as described by Rinttilä et al. [24], using the Q-PCR Core kit for SYBR Green I (Eurogentec, Seraing, Belgium) and primers Bacter140f and Bacter140r. The Q-PCR for *Bifidobacterium animalis lactis* was performed as described by Ventura et al. [25] using the primers (Bflact2 and Bflact5). All Q-PCR were performed with an ABI PRISM SDS 7000 Sequence Detection System (Applied Biosystems, Nieuwerkerk a/d Ijssel, the Netherlands).

### Blood parameters

Plasma insulin concentrations were determined using an ELISA kit (Mercodia, Uppsala, Sweden). The insulin resistance index was calculated by multiplying the area under the curve for glucose, and the area under the curve for insulin, calculated from −30 min until 15 min after glucose challenge [5], [26]. Concentrations of IL-6 and MCP-1 were determined in 15 µl of plasma using a multiplex immunoassay kit (Bio-Plex Cytokine Assay, Bio-Rad, Belgium) and measured using Luminex® technology (Bioplex®, Bio-Rad, Belgium). Adiponectin concentrations were determined using an ELISA kit designed to measure full-length mouse adiponectin levels in serum (Quantikine® Mouse adiponectin, R&DSystems).

Plasma triglycerides, cholesterol and non esterified fatty acid concentrations were measured using kits coupling enzymatic reaction and spectrophotometric detection of reaction end-products (Diasys Diagnostic and Systems, Holzheim, Germany). High density lipoprotein cholesterol (HDL-cholesterol) concentration was measured enzymatically after very low density lipoprotein (VLDL), chylomicrons and low density lipoprotein cholesterol (LDL-cholesterol) antibodies precipitation (Diasys Diagnostic and Systems, Holzheim, Germany). LDL was estimated by the Friedewald formula [27].

### Lipid analysis in the liver

Triglycerides and cholesterol were measured in the liver tissue after extraction with chloroform–methanol as described by Neyrinck et al. [28].

### Fatty acid synthase (FAS) activity

Homogenate of subcutaneous adipose tissue was performed in phosphate buffer (100 mg tissue/500 µl buffer). Cytosolic fractions were obtained after 2 successive centrifugations at 4°C (1000 g-15 min and 20000 g 30 min) The procedure described by Linn was used for the measurement of FAS activity in cytosolic fractions [29]. Protein was determined by the Bradford method.

### Adipose tissue morphometry

Number of adipocytes per microscopic field (density) was estimated on paraffin-embedded hematoxylin-stained eosin- counterstained sections of subcutaneous adipose tissue using the image analyzer software (Motic Image Plus 2.0 ML), as previously described [30].

### Fatty acid profile analysis in adipose tissue

Fatty acid analysis, including conjugated linoleic acid (c9, t11 CLA), in subcutaneous adipose tissue was performed according to the method described by Dewulf et al [30].

### Expression of selected genes in tissues

Total RNA was isolated using the TriPure isolation reagent kit (Roche Diagnostics Belgium, Vilvoorde). cDNA was prepared by reverse transcription of 1 µg total RNA using the Kit Reverse transcription System (Promega, Leiden, The Netherlands). Real-time PCRs were performed with the StepOnePlus™ real time PCR system and software (Applied Biosystems, Den Ijssel, The Netherlands) using Mesa Fast qPCR™ (Eurogentec, Seraing, Belgium) for detection according to the manufacturer's instructions. RPL19 RNA was chosen as housekeeping gene. Primer sequences for the targeted mouse genes are available on request (**audrey.neyrinck@uclouvain.be**). All samples were run in duplicate in a single 96-well reaction plate and data were analysed according to the 2-ΔCT method [31]. The identity and purity of the amplified product was checked through analysis of the melting curve carried out at the end of amplification.

### Statistical analysis

Results are presented as mean ± SEM. Statistical analysis was performed by ANOVA followed by *post hoc* Tuckey's multiple comparison test (GraphPad Software, San Diego, CA, USA); p<0.05 was considered as statistically significant. Correlations between parameters were assessed by Pearson's correlation test; correlations were considered significant as follows: *p<0.01, **p<0.001, ***p<0.0001 with the absolute value of Pearson r>0.5.

## Results

### Supplementation with arabinoxylan modified the gut microbiota composition

**Figure 1:**
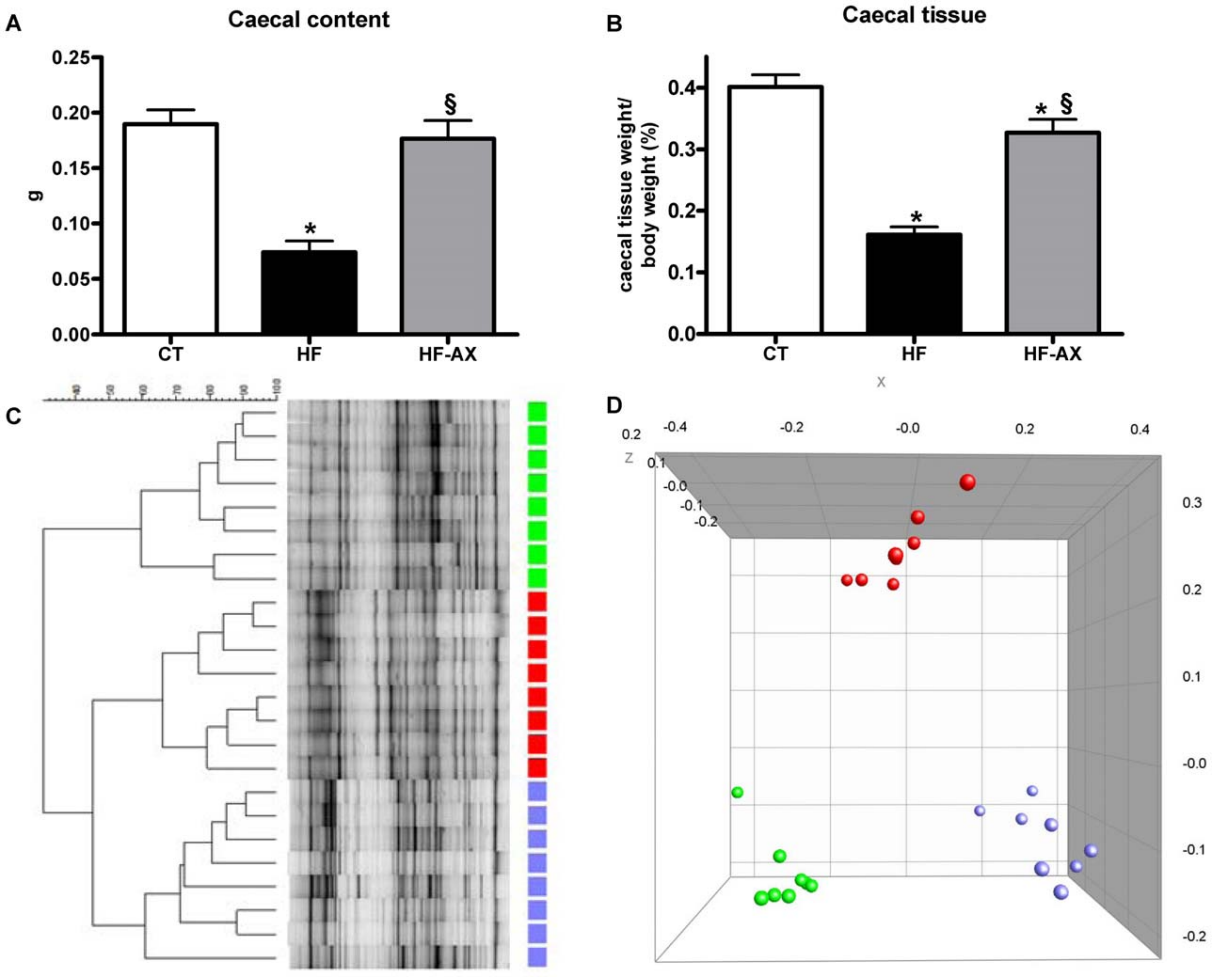
Caecum weight, DGGE fingerprints and PCA analysis in the caecal content. Caecal content (A) and caecal tissue (B) weight. Denaturing gradient gel electrophoresis (DGGE) fingerprint patterns of the caecal microbial community; the DGGE profiles were constructed using primers for total bacteria (C). Principal Coordinate Analysis (PCA) was used to explore the similarity within a composite data set consisting of DGGE fingerprints of total bacteria, bifidobacteria, lactobacilli and the *Bacteroides-Prevotella* spp. cluster (D). Mice were fed a standard (CT, green symbols), a high fat diet (HF, red symbols) or a high fat diet supplemented with 10% arabinoxylan (HF-AX, blue symbols) for 4 weeks. *p<0.05 versus CT and ^§^p<0.05 versus HF (ANOVA).

**Figure 2:**
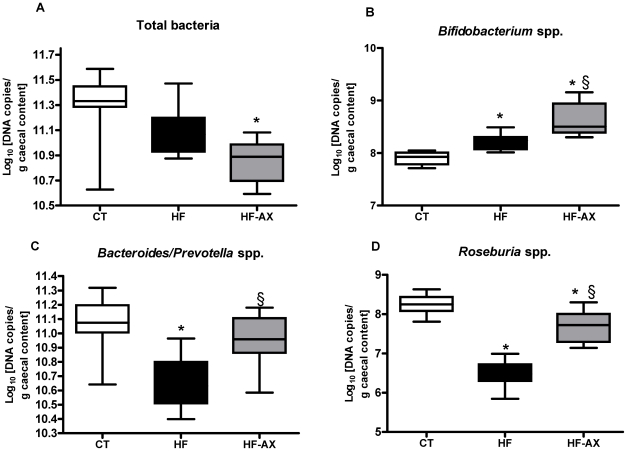
Bacterial quantification per gram of caecal content. Caecal bacterial content of total bacteria (A), *Bifidobacterium* spp. (B), *Bacteroides-Prevotella* spp. (C), *Roseburia* spp. (D). Bacterial quantities are expressed as Log10 (bacterial cells/ g caecal content wet weight). Mice were fed a standard (CT), a high fat diet (HF) or a high fat diet supplemented with 10% arabinoxylan (HF-AX) for 4 weeks. *p<0.05 versus CT and ^§^p<0.05 versus HF (ANOVA).

HF feeding decreased both the caecal content weight and tissue weight as compared to the control condition (Figures 1A and 1B). Clustering of the DGGE fingerprints for total bacteria (Figure 1C) indicated a separate cluster for the CT and HF groups. PCA analysis of combined DGGE fingerprints of total bacteria, bifidobacteria, lactobacilli and *Bacteroides-Prevotella* spp. shows distinct clusters between the HF and CT groups (Figure 1D). This was further confirmed following Q-PCR analyses of different bacterial groups (Figure 2 and Figure S1). HF diet induced a drop in *Roseburia* spp. and *Bacteroides-Prevotella* spp. numbers. Conversely, the number of bifidobacteria per gram of caecum content was significantly higher in the HF group, as compared to control mice (Figure 2B).

AX supplementation induced caecal enlargement (Figures 1A and 1B) and a profound shift in the microbial community in comparison with the HF group, as shown by the distinct clustering in the DGGE profile and PCA analysis (Figures 1C and 1D). AX supplementation restored the HF diet-induced microbial community changes as shown by the significant increase in *Bacteroides–Prevotella* spp. and *Roseburia* spp. (Figures 2C and 2D). Moreover, AX induced a specific increase in bifidobacteria (Figure 2B), in particular *bifidobacterium animalis ssp lactis* (expressed as Log10 [DNA copies/g caecal content]: 5,67±0,07, 6,03±0,07*, and 6,37±0,06*§ for CT, HF and HF-AX, respectively; ANOVA, * p<0.05 versus CT and §p<0.05 versus HF).

### Supplementation with arabinoxylan modified the expression of markers related to gut barrier function

The mRNA levels of both ZO-1 and occludin, which are tight-junction proteins, were measured in the jejunum, as well as the mRNA level of proglucagon, which is the precursor of the intestinotrophic peptide GLP-2, known to reduce gut permeability [4]. HF feeding did not modify the expression of these genes (Figure S2). Interestingly, AX supplementation significantly increased the mRNA levels for both tight-junction proteins and proglucagon expression, as compared to the HF and/or CT groups.

### Supplementation with arabinoxylan decreased body weight gain and fat mass development

**Figure 3:**
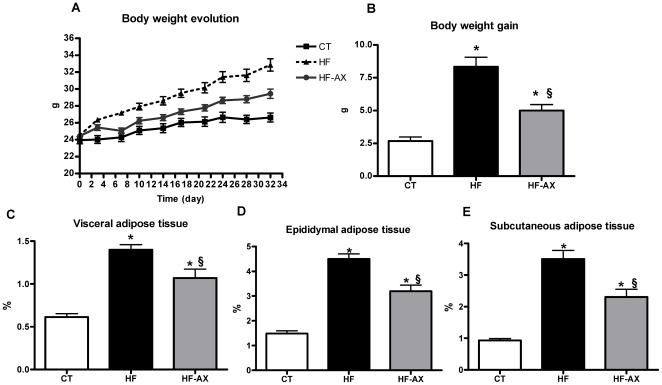
Body weight and fat mass. Body weight evolution (A), body weight gain (B), visceral (C), epididymal (D), and subcutaneous (E) adipose tissue weight (% versus body weight) of mice fed a standard (CT), a high fat diet (HF) or a high fat diet supplemented with 10% arabinoxylan (HF-AX) for 4 weeks. *p<0.05 versus CT and ^§^p<0.05 versus HF (ANOVA).

The mice fed with HF-AX did not gain weight as rapidly as HF-fed mice (Fig. 3A). In fact, AX supplementation decreased body weight gain by about 40% as compared to HF (fig. 3B). Moreover, AX treatment induced a lower fat mass development as shown by the weight of epididymal, subcutaneous and visceral adipose tissues (Figures 3C, 3D and 3E). This effect could not be explained by changes in energy intake since the total calorie intake (for 4 mice, n = 2) was not different between the HF and HF-AX groups (1579±30 kcal and 1539±15 kcal, respectively).

### Supplementation with arabinoxylan improved insulinoresistance index and cholesterol levels

HF feeding induced fasting hyperglycemia and hyperinsulinemia, and increased the insulin resistance index upon OGTT, as compared to mice fed normal chow diet (Table 1). Surprisingly, adiponectin level was not modified after HF treatment. HF diet feeding induced hepatic triglyceride accumulation (by about 28%) and increased serum total, LDL- and HDL-cholesterol levels without affecting serum triglycerides and non-esterified fatty acids (Table 1). The HF-induced hypercholesterolemia was associated to a higher hepatic content of free cholesterol.

**Table 1:** Blood and hepatic parameters.

	CT	HF	HF-AX
*Serum*			
- fasting glycemia (mM)	7.49±0.28	9.58±0.41*	8.44±0.41
- fasting insulinemia (mM)	83.9±12.6	226.3±32.2*	156.1±16.6*
- insulin resistance index	30030±963	113500±19440*	71990±7423*§
- adiponectin (pg/ml)	9.36±0.40	9.38±0.46	7.76±0.55 *§
- triglycerides (mM)	1.47±0.11	1.71±0.13	1.54±0.11
- non esterified fatty acids (mM)	0.27±0.03	0.31±0.03	0.29±0.04
- total cholesterol (mM)	1.75±0.06	3.00±0.03 *	2.41±0.06 *§
- LDL-cholesterol (mM)	0.31±0.05	1.22±0.08 *	0.90±0.06 *§
- HDL-cholesterol (mM)	0.78±0.02	1.00±0.03 *	0.82±0.02 §
*Liver lipid content, nmol/mg protein*			
- triglycerides	60.9±3.0	77.8±5.3 *	66.1±8.0
- total cholesterol	41.1±4.1	74.2±11.0 *	73.2±10.5 *
- free cholesterol	27.0±2.2	40.3±4.6 *	27.8±1.3 §
- esterified cholesterol	14.1±3.2	33.9±6.6	45.5±9.3 *

Mice were fed a standard diet (CT), a high fat diet (HF) or a high fat diet supplemented with 10% arabinoxylan (HF-AX) for 4 weeks * p<0.05 versus CT and ^§^p<0.05 versus HF (ANOVA). LDL, low density liprotein; HDL, high density lipoprotein.

AX did not change significantly fasting glycemia or insulinemia. Interestingly, AX feeding improved the insulin resistance index as compared to the HF group. However, we observed a lower concentration of adiponectin after AX supplementation (Table 1). AX supplementation furthermore decreased hypercholesterolemia (both LDL- and HDL-cholesterol) and also led to a shift from hepatic free cholesterol towards esterified cholesterol, without changing total cholesterol or triglyceride level in the tissue (Table 1).

### Supplementation with arabinoxylan increased the proportion of rumenic acid in the subcutaneous adipose tissue reflecting changes in gut bacterial metabolism

The fatty acid profile in adipose tissue is influenced by that of the dietary lipids [32], [33]. In addition, specific fatty acids, such as vaccenic acid (C18:1 t11) and conjugated linoleic acids, the major being the rumenic acid (C18:2 c9,t11), may be formed upon bacterial metabolism (biohydrogenation) of linoleic acid in the intestine [34]. Therefore, we analyzed the fatty acid patterns in subcutaneous adipose tissue of mice (Table 2). HF feeding induced drastic changes in the fatty acid composition of the adipose tissue with a higher proportion of monounsaturated fatty acids (MUFA), at the expence of both polyunsaturated fatty acids (PUFA) and saturated fatty acids (SFA). Interestingly, HF feeding induced a significant increase in both vaccenic acid and rumenic acid. Supplementation with AX significantly decreased the proportion of palmitic acid (C16:0) whereas it further increased the rumenic acid content as compared to the HF treatment.

**Table 2:** Fatty acid pattern of the subcutaneous adipose tissue from mice fed a standard diet (CT), a high fat diet (HF) or a high fat diet supplemented with 10% arabinoxylan (HF-AX) for 4 weeks.

	CT			HF			HF-AX		
**SFA**	**28.197**	**±**	**0.614**	**25.998**	**±**	0.180*	**25.010**	**±**	0.322*§
C6:0	0.017	±	0.002	0.007	±	0.001*	0.006	±	0.001*
C8:0	0.009	±	0.001	0.004	±	0.001*	0.004	±	0.001*
C10:0	0.045	±	0.003	0.039	±	0.003	0.040	±	0.002
C12:0	0.119	±	0.009	0.080	±	0.006*	0.082	±	0.004*
C13:0	0.014	±	0.001	0.010	±	0.001*	0.011	±	0.001
C14:0	1.563	±	0.057	1.010	±	0.017*	0.997	±	0.024*
C15:0 iso	0.013	±	0.001	0.003	±	0.000*	0.003	±	0.001*
C15:0 anteiso	0.013	±	0.001	0.003	±	0.001*	0.004	±	0.001*
C15:0	0.169	±	0.005	0.106	±	0.001*	0.107	±	0.003*
C16:0 iso	0.133	±	0.003	0.067	±	0.003*	0.069	±	0.003*
C16:0	22.422	±	0.459	19.844	±	0.138*	18.631	±	0.338*§
C17:0 iso	0.150	±	0.003	0.143	±	0.003	0.138	±	0.003*
C17:0 anteiso	0.683	±	0.015	0.737	±	0.014	0.798	±	0.032*
C17:0	0.181	±	0.005	0.250	±	0.004*	0.248	±	0.004*
C18:0	2.525	±	0.136	3.631	±	0.088*	3.791	±	0.177*
C20:0	0.140	±	0.005	0.065	±	0.004*	0.080	±	0.007*
**MUFA**	**44.442**	**±**	**0.260**	**48.981**	**±**	0.133*	**49.778**	**±**	0.438*
C14:1 C9	0.153	±	0.008	0.062	±	0.010*	0.052	±	0.003*
C16:1C9	8.631	±	0.207	4.727	±	0.160*	4.407	±	0.229*
C18:1T9	0.040	±	0.003	0.224	±	0.008*	0.240	±	0.007*
C18:1T11	0.055	±	0.004	0.192	±	0.007*	0.194	±	0.005*
C18:1C9	32.341	±	0.214	41.253	±	0.143*	42.283	±	0.593*
C18:1C11	3.222	±	0.049	2.520	±	0.019*	2.602	±	0.023*§
**PUFA**	**27.361**	**±**	**0.583**	**25.021**	**±**	0.164*	**25.211**	**±**	0.375*
C18:2C9C12	25.698	±	0.544	23.115	±	0.146*	23.436	±	0.294*
C18:3C9C12C15	0.859	±	0.032	1.081	±	0.026*	0.991	±	0.062
C18:2C9T11	0.090	±	0.005	0.171	±	0.002*	0.181	±	0.003*§
C20:3C11C14C17	0.013	±	0.001	0.061	±	0.001*	0.060	±	0.002*
C20:4C5C8C11C14	0.323	±	0.011	0.362	±	0.007	0.332	±	0.017
C20:5C5C8C11C14C17	0.041	±	0.005	0.020	±	0.001*	0.013	±	0.003*
C22:5C7C10C13C16C19	0.063	±	0.002	0.065	±	0.002	0.060	±	0.003
C22:6C4C7C10C13C16C19	0.264	±	0.012	0.141	±	0.003*	0.132	±	0.008*

Values are means ± SEM (g/100 g of identified fatty acid methyl esters). SFA, saturated fatty acids; MUFA, monounsaturated fatty acids; PUFA, polyunsaturated fatty acids.

### Supplementation with arabinoxylan reduced adipocyte size and decreased HF-induced gene expression in the subcutaneous adipose tissue

**Figure 4:**
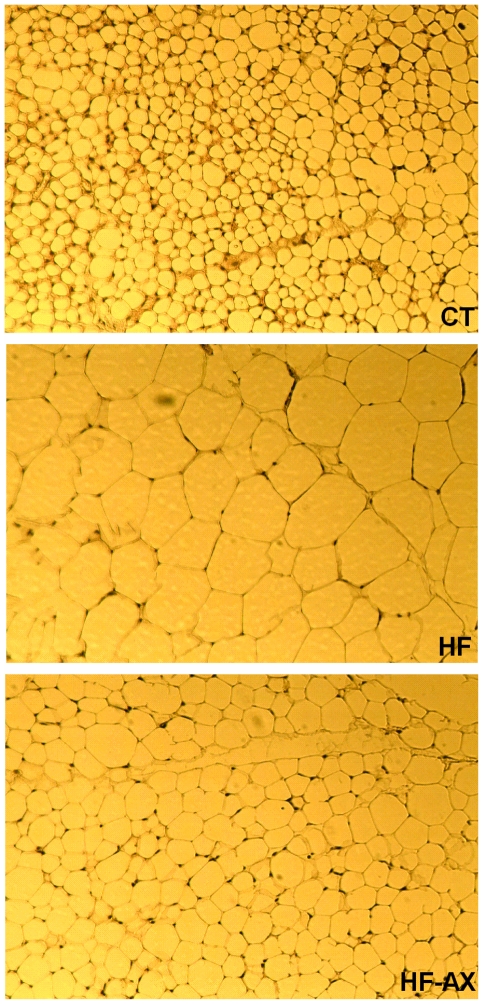
Histological pictures of subcutaneous adipose tissue. Mice were fed a standard diet (CT), a high fat diet (HF) or a high fat diet supplemented with 10% arabinoxylan (HF-AX) for 4 weeks.

**Figure 5:**
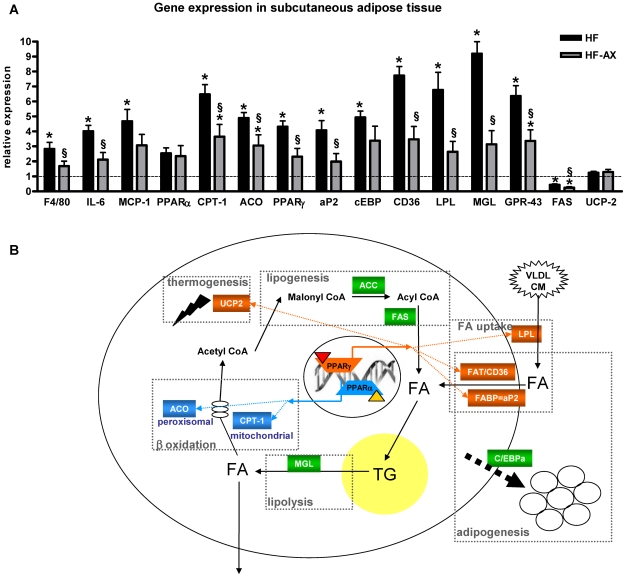
mRNA levels of key factors and metabolic network in the subcutaneous adipose tissue. Expression of genes involved in subcutaneous adipose tissue metabolism (A). Mice were fed a standard (CT), a high fat diet (HF) or a high fat diet supplemented with 10% arabinoxylan (HF-AX) for 4 weeks. Values are expressed relative to CT group (set at 1). *p<0.05 versus CT and ^§^p<0.05 versus HF (ANOVA). Genes that regulate metabolic processes in white adipose tissue (B); some of them are dependent on PPARα (blue) or PPARγ (orange) activation by an endogenous ligand. PPARγ, peroxisome proliferator-activated receptor γ; aP2, adipocyte fatty acid binding protein; C/EBPα, CCAAT enhancer binding protein α; GPR43, G protein-coupled receptor 43; LPL, lipoprotein lipase; CD-36, cluster of differenciation 36; FAS, Fatty acid synthase; ACC, AcylCoa carboxylase; PPARα, peroxisome proliferator-activated receptor-alpha ; CPT-1, carnitine palmitoyl transferase-1 ; ACO, AcylCoA oxydase; MGL, monoacylglycerol lipase; UCP-2, uncoupling protein-2; VLDL, very low density lipoprotein; CM, chylomicron; FA, fatty acids; TG, triglycerides.

Histological analysis revealed that the adipocyte size in subcutaneous adipose tissues were increased in the HF-fed mice versus controls, whereas the AX treatment normalized this parameter (Figure 4; adipocyte number per field: 339±49, 71±13* and 177±21*§ for CT, HF and HF-AX, respectively; ANOVA, * p<0.05 versus CT and §p<0.05 versus HF). HF feeding increased the expression of genes controlling inflammation (F4/80, IL-6, MCP-1), PPAR-α dependent-fatty acid oxidation (CPT-1, ACO), PPARγ-dependent differentiation and/or fatty acid uptake (C/EBPα, FAT/CD36, aP2, LPL), and lipolysis (MGL) (Figure 5). Furthermore, the mRNA content of GPR43 -a receptor activated by short-chain fatty acids and implicated in the regulation of lipolysis and adipocyte differentiation- was significantly increased upon HF feeding. Interestingly, the AX treatment hugely decreased the expression of the most of these genes in the subcutaneous tissue. Of particular interest, AX treatment decreased the serum concentrations of 2 inflammatory markers that were downregulated in the adipose tissue through AX treatment, namely IL6 (53.9±11.5 pg/ml and 21.9±7.7 pg/ml for HF and HF-AX groups, respectively; t test p<0.05) and MCP-1 (32.1±5.3 pg/ml and 12.3±2.4 pg/ml for HF and HF-AX groups, respectively; t test p<0.05). In addition, AX supplementation inhibited the expression of fatty acid synthase (FAS), a lipogenic enzyme that was already downregulated by the HF diet. In accordance with its expression, we confirmed that FAS activity was downregulated through AX supplementation in adipose tissue since its activity was significantly lower as compared to HF group (32.5±7.1, 25.5±7.5 and 7.1±1.3*§ for CT, HF and HF-AX respectively; ANOVA: *p<0.05 versus CT and ^§^p<0.05 versus HF). By contrast, it did not affect the expression of the uncoupling protein UCP-2.

Given that AX treatment decreased genes involved in fatty acid uptake, we decided to explore some markers of lipid metabolism in the liver and the muscle (Table S2). In the *vastus lateralis* muscle, there were no changes in mRNA expression of genes involved in fatty acid oxidation (ACO, PPARα, PPARδ, CPT1) between the groups. In the liver, HF feeding induced the expression of the genes encoding sterol regulatory element-binding protein-1c (SREBP-1c) and its downstream regulated protein FAS. AX supplementation did not modify significantly lipogenic gene expression but it lowered HF-induced PPARα expression with no consequences on genes involved in fatty acid oxidation (CPT1 or ACO). Of note, there was no change in the expression of genes involved in cholesterol metabolism, whatever the dietary treatments.

### Improved metabolism following arabinoxylan supplementation was correlated with the increase in bifidobacteria or the restoration of Roseburia spp. and Bacteroides/Prevotella spp

**Figure 6:**
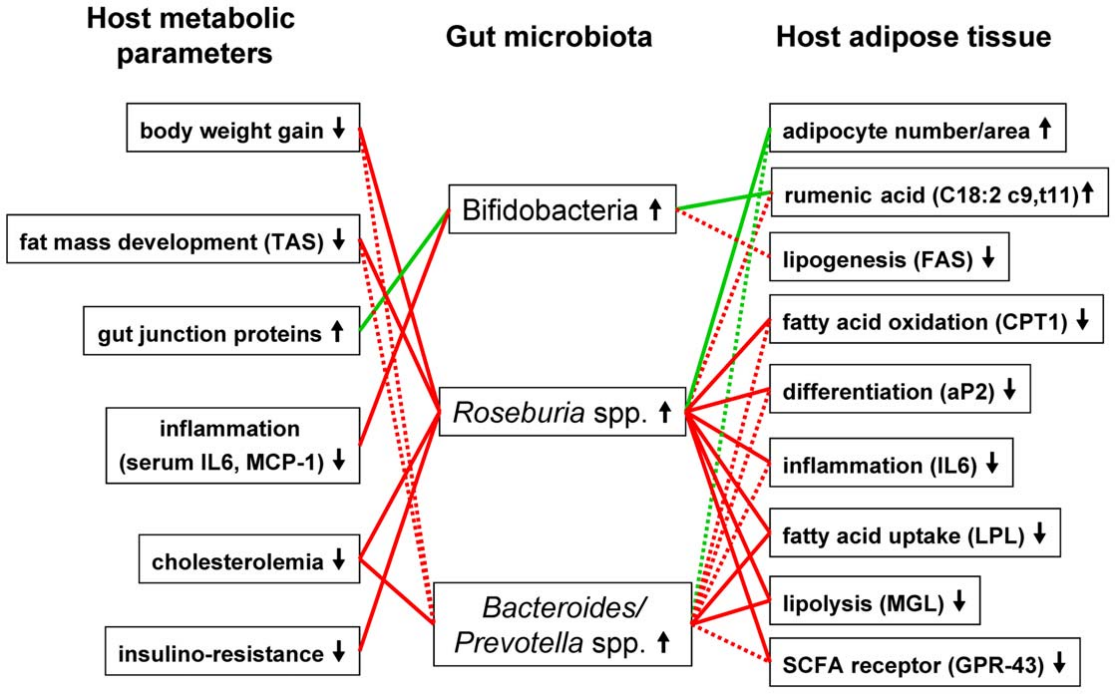
Interrelationship between gut microbiota composition and host metabolic parameters significantly modified by arabinoxylan supplementation. Green connections indicate a positive correlation (Pearson r>0.5), while red connections show correlations that are inverse (Pearson r<0.5). Solid lines represent significance with p<0.001 and shared lines represent significance with p<0.01. aP2, adipocyte fatty acid binding protein; GPR43, G protein-coupled receptor 43; IL6, interleukin 6; LPL, lipoprotein lipase; FAS, Fatty acid synthase; CPT-1, carnitine palmitoyl transferase-1 ; MCP-1, monocyte chemoattractant protein-1; MGL, monoacylglycerol lipase; SCFA, short chain fatty acid.

To determine whether modifications of the gut microbiota in mice fed AX were associated with an improvement in obesity and lipid metabolism, a correlation analysis was performed between bacteria and host metabolic parameters that were significantly affected through AX supplementation (Figure 6 and Table S3). The analysis revealed negative correlations (p<0.001) between *Roseburia spp*, and fat mass development, body weight gain, cholesterolemia, insulinoresistance index, and expression of several genes that mediate differentiation and/or fatty acid uptake (PPARγ, aP2, FAT/CD36, LPL, FIAF), fatty acid oxidation (CPT-1, ACO), short-chain fatty acid response (GPR43), and inflammation (IL6, F4/80) in the subcutaneous adipose tissue. Some of these metabolic parameters were also inversely correlated with the number of *Bacteroides/Prevotella* spp. (p<0.01) whereas none of these markers was significantly correlated with the number of total bifidobacteria. However, bifidobacteria levels were positively correlated with rumenic acid in the adipose tissue and with the mRNA levels of the tight junction proteins (ZO-1 and occludine) in the gut. The analysis revealed other interesting negative correlation with bifidobacteria such as the FAS activity/expression in the adipose tissue and circulating inflammatory markers (IL-6 and MCP-1).

## Discussion

Over the past 5 years, animal and human studies have revealed a remarkable microbial influence on host metabolism, energy utilization and storage, and metabolic diseases [1], [35]–[37]. A shift in the composition the murine gut microbiota, such as a decrease in Bacteroidetes or an increase in Firmicutes, was reported to be induced by dietary interventions in favour of a HF diet [3], [28], [38]. The microbial-related response of mice towards a HF diet, in terms of fat mass development and metabolic diseases, is dependent on the diet composition [39]. Here, we show that feeding mice with a HF diet decreases the dominant members of the mouse intestinal microbiota - *Bacteroides-Prevotella* ssp. from the Bacteroidetes phylum together with the specific populations of clostridial cluster XIVa, i.e. *Roseburia* spp from the Firmicutes phylum. However, it slightly increased the number of total bifidobacteria. The composition of the HF diet as compared to the standard diet -in term of dietary fiber (concentration and nature)- is probably responsible for the important changes in the parameters related to the gut fermentation (caecal content weight and microbial composition). Indeed, according to the manufacturer, the control diet contains 84% of cereal products that brings 4% of cellulose as dietary fibre without taking into account other non digestible/fermentable carbohydrates such as arabinoxylan, resistant starch, fructooligosaccharides present in cereal in particular in bran fractions [8]. In contrast, the dietary fibre of the HF diet is originating from cellulose BW200 (6.5%) which is poorly fermentable. Moreover, the HF diet has only maltodextrin and sucrose as digestible carbohydrate sources whereas the control diet has native starch coming from cereals, bran and remilling; a part of this starch being resistant to digestion and consequently entering in the caeco-colon as fermentable fraction (resistant starch). Interestingly, AX added to the HF diet led to an increase of bifidobacteria and in particular of *Bifidobacterium animalis subsp. Lactis*. AX also restored to control level, the bacterial populations in the caecal content which were decreased upon HF feeding, namely the gram negative *Bacteroides-Prevotella* spp. and the gram positive bacteria *Roseburia* spp.

In previous studies, we demonstrated that feeding rats with the inulin-type prebiotic (oligofructose) protected against liver triglyceride accumulation induced by fructose and that the lower lipogenic capacity of the liver could be the key event in this protection; indeed FAS activity remained significantly lower in oligofructose-fed rats [40]–[42]. More recently, we found that a selective change in gut microbiota composition through inulin-type prebiotic treatment improves gut barrier functions through a GLP-2-dependent mechanism during obesity and diabetes [4]. As shown for oligofructose, the bifidogenic effect of AX demonstrated here, were accompanied by a decrease in FAS activity in the adipose tissue. Moreover, we observed an increase in both tight junction proteins and proglucagon expression after AX supplementation, suggesting an increase in gut barrier-related functions as a physiological consequence of the changes in the composition of gut microbiota. Of particular interest, we observed lower levels of inflammatory markers in the serum due to AX supplementation that could be the result of improvement of gut barrier functions as suggested by Cani et al. [3], [5]. Interestingly, those metabolic changes were correlated with the number of bifidobacteria in the caecal content confirming prebiotic properties of AX.

Here, we demonstrate for the first time that a prebiotic approach is also able to change the occurrence of PUFA metabolites in the white adipose tissue. Such an effect was previously obtained with a probiotic strategy, leading to modifications of fatty acid pattern in the adipose tissue (including higher concentrations of the n−3 fatty acids eicosapentaenoic acid and docosahexaenoic acid) [43]. AX increases the amount of a linoleic acid metabolite, namely rumenic acid which belong to the conjugated linoleic acid (CLA) family. CLAs comprise a set of positional (eg, 9,11; 10,12; 11,13) and geometric (cis or trans) isomers of linoleic acid with conjugated double bonds. CLA isomers have been shown to exert a variety of biological activities and some of them have been shown to exert anti-obesity effects [44]. Commensal bifidobacteria from the mammalian gut have been shown to generate CLA, predominantly rumenic acid -the c9,t11 isomer from free linoleic acid- whereas *Roseburia* spp. formed either vaccenic acid (18:1 t11) or a 10-hydroxy-18:1, two precursors of rumenic acid [34], [45], [46]. We observed a higher proportion of rumenic acid upon HF feeding alone, probably due to a higher concentration of its substrate (linoleic acid) in the HF diet versus control diet. AX supplementation further increased the rumenic acid proportion in host adipose tissue, suggesting a specific linoleic acid metabolism by CLA-producing bacteria such as bifidobacteria and indirectly *Roseburia* spp, which are both promoted upon AX feeding.

To date, only a few studies have investigated whether the consumption of wheat-derived AX could have potential beneficial effects for health. We show here that, in parallel to changes in gut bacterial population, the specific concentrate of water-extractable high molecular weight AX significantly decreased body weight gain and fat mass development in a model of HF diet-induced obesity. In addition, AX supplementation counteracted both the HF-induced hypercholesterolemia (total, LDL- and HDL-cholesterol) and the higher content of free cholesterol in the liver upon HF feeding. The hypocholesterolemic effect of AX has previously been observed *in vivo* in rats, an effect attributed to a decreased dietary cholesterol absorption and an increased fecal excretion of cholesterol and bile acids, leading -as a metabolic signature- to an increased expression of HMG-CoA reductase [13]. We may not exclude that the higher production of propionate upon AX fermentation could be implicated in the decrease in hepatic cholesterol synthesis and hypocholesterolemic effect [19]. However, the fact that we were unable to see any changes in hepatic HMG-CoA reductase expression is not in favor of this mechanism in our study.

The histological analysis of the subcutaneous adipose tissue showed that the adipocytes were larger upon the HF treatment and became smaller when the HF diet was combined with AX. PPARs are vital for adipogenesis (adipocyte differentiation), fatty acid uptake, lipogenesis and fatty acid oxidation (see fig. 5 and [47], [48]). PPARγ is abundantly expressed in adipose tissue, where it is a key regulator of adipocyte differentiation, whereas PPARα governs fatty acid oxidation [47]. Importantly, antagonism of PPARγ using a synthetic ligand suppresses the increased adiposity observed in HF induced obesity [47]. Recently, we have shown that inulin-type fructans with prebiotic properties counteract PPARγ-related adipogenesis in the white adipose tissue of HF fed mice [30]. Here, we demonstrate that the supplementation of the specific AX concentrate is also able to decrease the expression of most of the HF-induced metabolic genes in subcutaneous adipose tissue, in particular genes depending on PPARs-activation. Indeed, AX down-regulated genes involved in adipocyte differentiation, fatty acid uptake, fatty acid oxidation, lipolysis while it decreased fatty acid synthesis (expression and activity) that was already downregulated upon HF-feeding. Although we can attribute the antiobesity effect of AX (lower fat mass development) to a lower fatty acid synthesis (FAS expression and activity) or fatty acid uptake in white adipose tissue (downregulation of lipoprotein lipase, fatty acid translocase/CD36 and fatty acid binding protein (aP2)), we may not exclude an effect of AX on lipid absorption through either its intrinsic capacity of fat binding or modulation of pancreatic lipase activity [49]. Chitosan –coming from chitin- has the capacity to bind fatty acids and to increase the lipid content in the caecum [50], [51]. This mechanism seems also to be involved in the improvement of HF-induced metabolic alterations by AX. Indeed, the proportion of lipid accumulated in the caecum versus the lipid ingested during a 12 h-feeding period was increased due to AX or chitosan supplementation in the HF diet (figure S3A). Furthermore, in this experiment, AX or chitosan supplementation increased both the fatty acid content and the cholesterol content in the caecum of mice fed a HF diet (figure S3B and S3C). Although the host adiposity changes and hypocholesterolemic effect of AX could be the result of its direct fat binding capacity leading to fat leakage in faecal matter, we may not exclude other potential mechanisms. The study of VO2 and VCO2 by indirect calorimetry at rest and during physical activity could constitute one interesting perspective to investigate further. In fact, we postulate that the modulation of gut microbiota induced by AX supplementation observed in the present study, was involved in its anti-obesity action as well as in its cholesterol-lowering effects. This hypothesis is supported by the fact that the number of bacteria from the clostridial cluster XIVa, such as *Roseburia* spp. -and *Bacteroides-Prevotella* spp. to a lesser extent- showed inverse correlations with important markers of obesity and host lipid metabolism (fat mass development, body weight gain, cholesterolemia, and expression of several genes mediating differentiation and/or fatty acid uptake, fatty acid oxidation, short chain fatty acid response and inflammation in the subcutaneous adipose tissue). Recently, Martinez and coworkers [52] provided evidence that modulation of the gut microbiota-host metabolic interrelationship by dietary intervention has the potential to improve mammalian cholesterol homeostasis, which has relevance for cardiovascular health. Several mechanisms could explain how the gut microbiota may affect host lipid metabolism since gut bacteria are able 1) to regulate chylomicron formation and lipid uptake by affecting gut transit time and bile salt metabolism (modulation of the enterohepatic circulation of bile acids) [1], [53]; 2) to ferment complex polysaccharides into short chain fatty acids that may act either as lipogenic substrates in the liver or an inhibitor of cholesterologenesis and lipogenesis from acetate [54]; 3) to suppress the expression of fasting-induced adipose factor (FIAF) in the intestinal mucosa, a factor able to increase lipoprotein lipase (LPL) dependent triglyceride storage in adipose tissue and to reduce serum triglyceride level [1], [55], [56]. FIAF is indeed an important regulator of lipid metabolism and has been shown to increase total cholesterol and high-density lipoprotein (HDL) cholesterol levels when over-expressed in transgenic mice [57]. This last mechanism could be involved in the hypocholesterolemic effect of AX since FIAF expression in white adipose tissue was decreased after AX supplementation in the HF diet.

Having a significant amount of excess body fat is a major problem that increases the risk of developing insulin resistance and type 2 diabetes. We have shown that the specific AX concentrate decreased HF-induced hyperglycemia and hyperinsulinemia, whereas its supplementation improved glucose tolerance after a glucose load (data not shown) but these effects were not significant. However, it is clear that the insulin resistance index was improved due to AX treatment as compared to the HF group, suggesting that the anti-obesity effect of AX was accompanied by a beneficial effect on insulin sensitivity. This result is in accordance with a clinical study demonstrating that consumption of the same AX concentrate for 6 weeks improved postprandial glycemia and insulinemia in overweight subjects with impaired glucose tolerance [16].

In conclusion, this study has shown in a 4-week study in mice, that supplementation of a concentrate of water-extractable high molecular weight AX in the diet counteracted HF-induced gut dysbiosis with a major effect on bacteria from clostridial cluster XIVa (restoration of *Roseburia* spp.), gram negative *Bacteroides-Prevotella* spp. and bifidobacteria (in particular, higher caecal content in *Bifidobacterium animalis subsp. Lactis*). This prebiotic effect was accompanied by a lower circulating inflammatory markers and by an increase in both tight junction markers and rumenic acid (18:2 c9,t11) in white adipose tissue. This last phenomenon reflects the influence of gut bacterial metabolism on host tissue. The gut microbiota changes due to the AX treatment were accompanied by an improvement of obesity and lipid-lowering effects together with an improvement of insulin resistance markers in HF diet-induced obesity. In addition, AX supplementation was able to decrease a number of PPARs- dependent genes within white adipose tissue, without inducing fat accumulation in the liver. Those metabolic effects may be related to the intrinsic fat binding capacity of AX. However, since it is known that gut bacteria could influence host lipid metabolism, we postulate that both hypocholesterolemic and anti-obesity effects conferred by AX are related, at least in part, to changes in gut microbiota.

Even if a direct extrapolation of the present study to human is still questionable due to differences in digestive tract structure and in gut microbiota, our results suggest that water extractable high molecular weight AX that is predominantly found in wheat endosperm can confer positive health impacts through gut microbiota modulation and may be a natural alternative in the prevention of obesity and cardiovascular diseases.

## Supporting Information

Figure S1 **Bacterial quantification in the caecum.** Caecal bacterial content of total bacteria (A), *Bifidobacterium* spp. (B), *Bacteroides-Prevotella* spp. (C) and *Roseburia* spp. (D). Bacterial quantities are expressed as Log10 (bacterial cells/ total caecal content wet weight). Mice were fed a standard diet (CT), a high fat diet (HF) or a high fat diet supplemented with 10% arabinoxylan (HF-AX) for 4 weeks.*p<0.05 versus CT and ^§^p<0.05 versus HF (ANOVA).
(TIF)

Figure S2 **mRNA levels of key markers in jejunum related to gut barrier function.** Mice were fed a standard (CT), a high fat diet (HF) or a high fat diet supplemented with 10% arabinoxylan (HF-AX) for 4 weeks. Values are expressed relative to CT group (set at 1). *p<0.05 versus CT and ^§^p<0.05 versus HF (ANOVA). ZO-1, zonula occludens-1.
(TIF)

Figure S3 **Analysis of fat binding capacity of arabinoxylan (AX) in vivo.** Eighteen male C57bl6/J mice (10 week old) were housed in groups of 3 per cage in a controlled environment with free access to HF diet. After 3 days for acclimatisation, the mice were divided into 3 groups (n = 6/group): a group fed with a HF diet, a group fed the same HF diet supplemented with 10% AX (HF-AX) and a group fed with the HF diet supplemented with 10% chitosan (KiOnutrime-Cs™ from KitoZyme sa, Belgium, HF-Cs). Food intake was recorded and mice were killed 12 h after access to the diets. Lipid content, fatty acids and cholesterol concentration in the caecal content were determined as previously described [50]. Proportion of caecal lipids versus ingested lipids (A), caecal pool of fatty acids (B) and caecal pool of cholesterol (C); *p<0.05 versus HF (ANOVA).
(TIFF)

Table S1 **The full composition of the control diet and the high fat diet.**
(DOC)

Table S2 **Gene expression in the liver and in the muscle of mice fed a standard diet (CT), a high fat diet (HF) or a high fat diet supplemented with 10% arabinoxylan (HF-AX) for 4 weeks.**
(DOC)

Table S3 **Analysis of correlation between the number of bacteria in the caecal content (expressed as Log10 (bacterial cells/ total caecal content wet weight) with metabolic parameters that were significantly affected by AX treatment.**
(DOC)
