# Supplementary figures and images for: Prebiotic Effects of Wheat Arabinoxylan Related to the Increase in Bifidobacteria, Roseburia and Bacteroides/Prevotella in Diet-Induced Obese Mice
Source: PLoS One. 2011 Jun 9;6(6):e20944. doi: 10.1371/journal.pone.0020944 (PMC3111466; doi:10.1371/journal.pone.0020944)

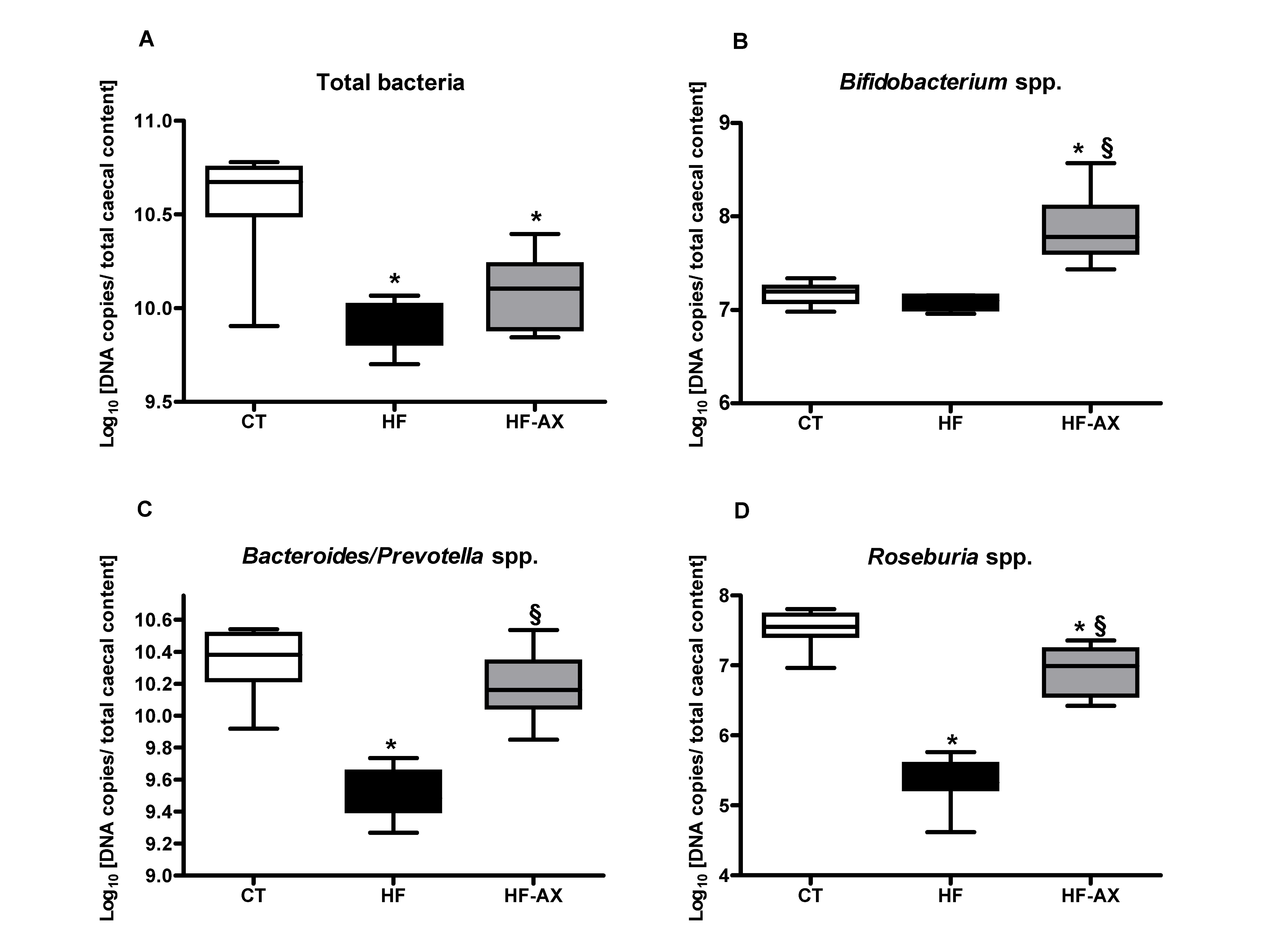

Supplement: Figure S1 — Bacterial quantification in the caecum. Caecal bacterial content of total bacteria (A), Bifidobacterium spp. (B), Bacteroides-Prevotella spp. (C) and Roseburia spp. (D). Bacterial quantities are expressed as Log10 (bacterial cells/ total caecal content wet weight). Mice were fed a standard diet (CT), a high fat diet (HF) or a high fat diet supplemented with 10% arabinoxylan (HF-AX) for 4 weeks.*p<0.05 versus CT and §p<0.05 versus HF (ANOVA). (TIF) [file pone.0020944.s001.tif]

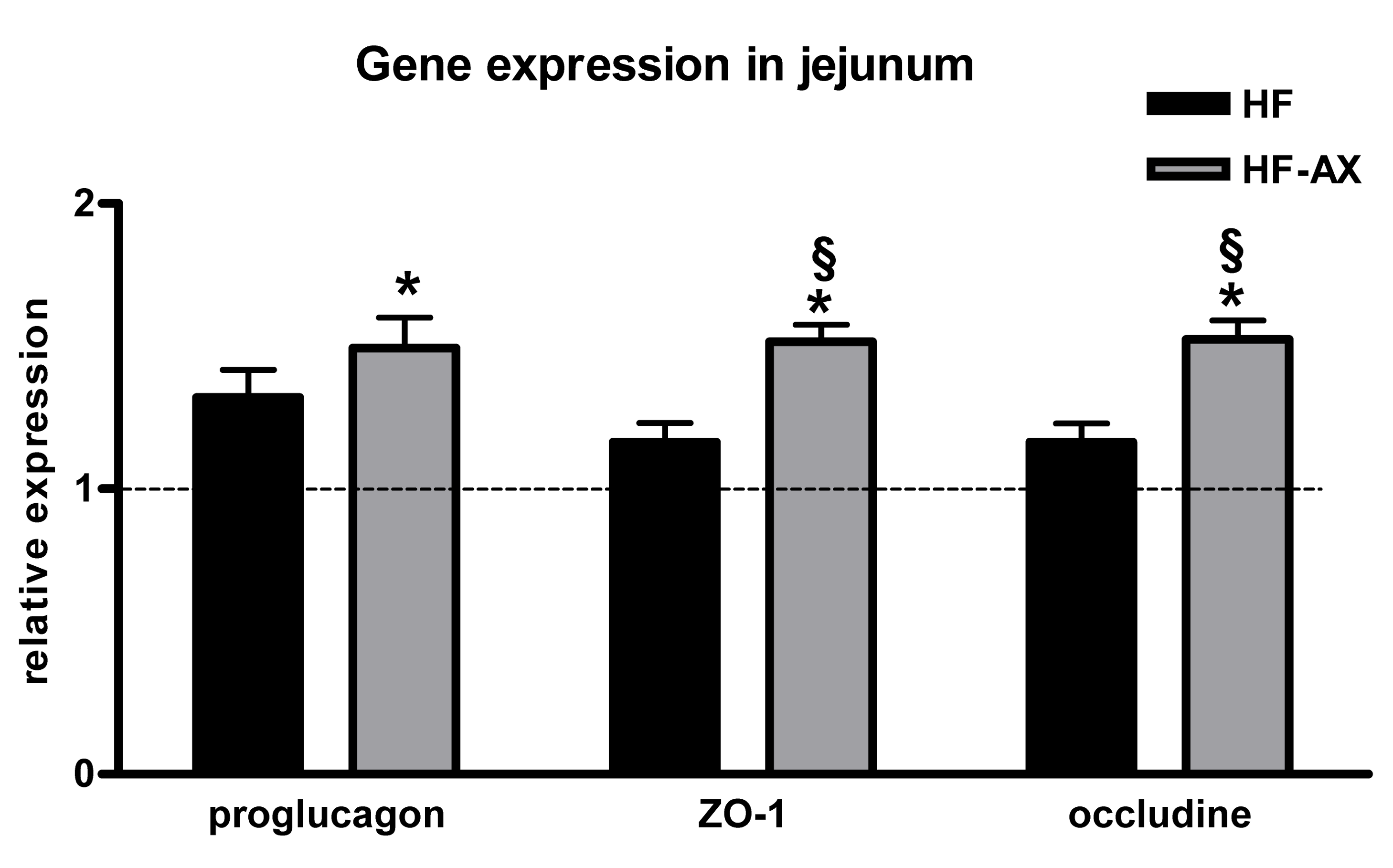

Supplement: Figure S2 — mRNA levels of key markers in jejunum related to gut barrier function. Mice were fed a standard (CT), a high fat diet (HF) or a high fat diet supplemented with 10% arabinoxylan (HF-AX) for 4 weeks. Values are expressed relative to CT group (set at 1). *p<0.05 versus CT and §p<0.05 versus HF (ANOVA). ZO-1, zonula occludens-1. (TIF) [file pone.0020944.s002.tif]

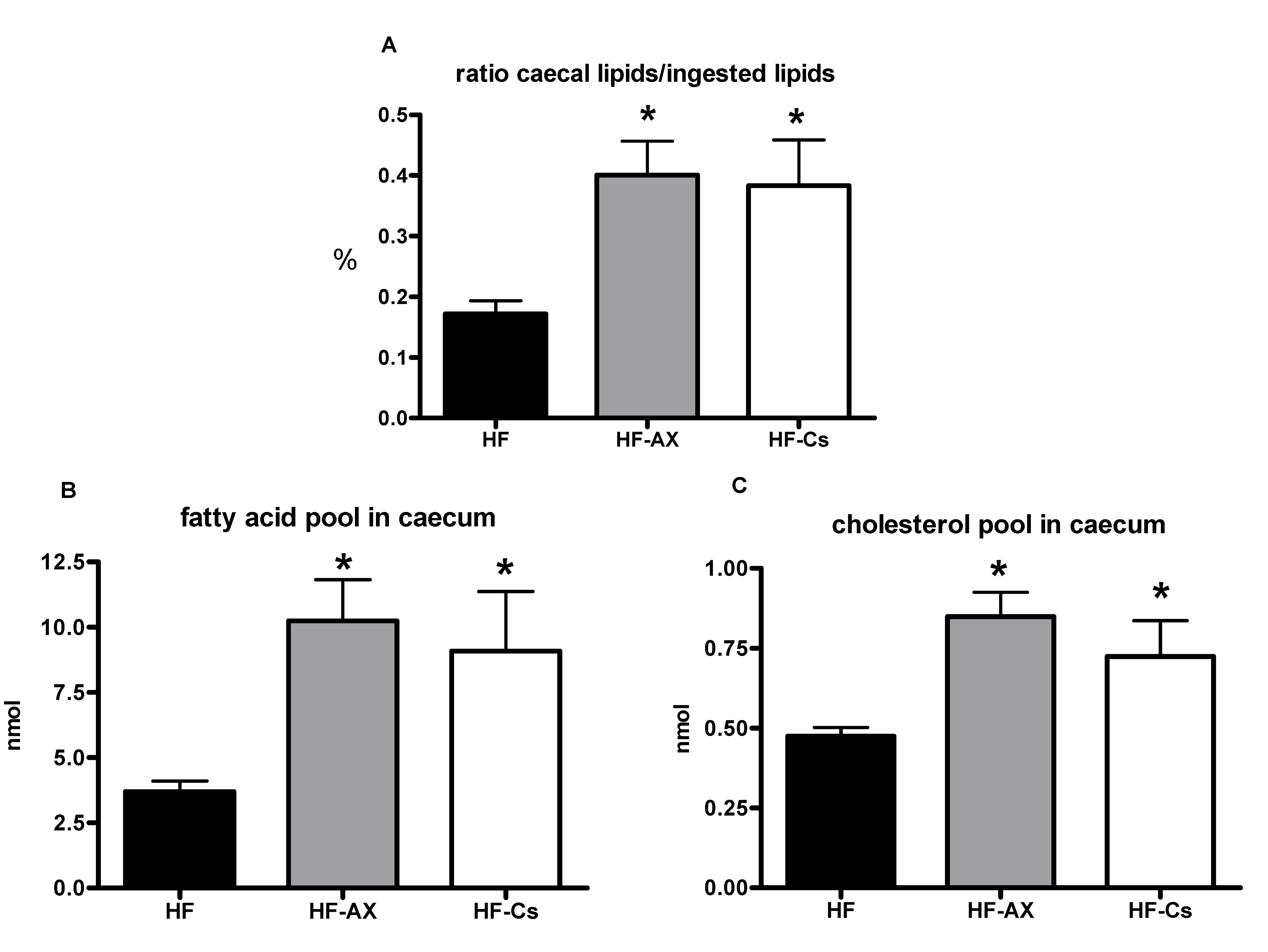

Supplement: Figure S3 — Analysis of fat binding capacity of arabinoxylan (AX) in vivo. Eighteen male C57bl6/J mice (10 week old) were housed in groups of 3 per cage in a controlled environment with free access to HF diet. After 3 days for acclimatisation, the mice were divided into 3 groups (n = 6/group): a group fed with a HF diet, a group fed the same HF diet supplemented with 10% AX (HF-AX) and a group fed with the HF diet supplemented with 10% chitosan (KiOnutrime-Cs™ from KitoZyme sa, Belgium, HF-Cs). Food intake was recorded and mice were killed 12 h after access to the diets. Lipid content, fatty acids and cholesterol concentration in the caecal content were determined as previously described [50]. Proportion of caecal lipids versus ingested lipids (A), caecal pool of fatty acids (B) and caecal pool of cholesterol (C); *p<0.05 versus HF (ANOVA). (TIFF) [file pone.0020944.s003.tiff]
