# Supplementary material for: Prebiotic Effects of Wheat Arabinoxylan Related to the Increase in Bifidobacteria, Roseburia and Bacteroides/Prevotella in Diet-Induced Obese Mice
Source: PLoS One. 2011 Jun 9;6(6):e20944. doi: 10.1371/journal.pone.0020944 (PMC3111466; doi:10.1371/journal.pone.0020944)
Supplement: Table S1 — The full composition of the control diet and the high fat diet. (DOC) [file pone.0020944.s004.doc]

|  | AO4 (Safe) | | HF (D12492, Research Diets) | |
| --- | --- | --- | --- | --- |
| Ingredients (g/100g) | fish protein  plant protein  vitamin mix  cereals (bran,remilling) | 4  8  4.1  83.9 | Casein, 80 Mesh  L-Cystine  Corn Starch  Maltodextrin 10  Sucrose  Cellulose, BW200  Soybean Oil  Lard  Mineral Mix S10026  DiCalcium Phosphate  Calcium Carbonate  Potassium Citrate.1H_2_O  Vitamin Mix V10001  Choline Bitartrate  FD&C Red Dye | 25.84  0.39  0.00  16.15  8.89  6.46  3.23  31.66  1.29  1.68  0.71  2.13  1.29  0.26  0.01 |
| Protein (%) | 16.1 | | 26.2 | |
| Carbohydrate (%)  - amidon  - saccharose  - maltodextrin  - cellulose | 60  45.8  2  /  3.9 | | 26.3  8.5  20.1  11.7  5.8 | |
| Fat (%) | 3.1 | | 34.9 | |
