# Supplementary material for: Prebiotic Effects of Wheat Arabinoxylan Related to the Increase in Bifidobacteria, Roseburia and Bacteroides/Prevotella in Diet-Induced Obese Mice
Source: PLoS One. 2011 Jun 9;6(6):e20944. doi: 10.1371/journal.pone.0020944 (PMC3111466; doi:10.1371/journal.pone.0020944)
Supplement: Table S2 — Gene expression in the liver and in the muscle of mice fed a standard diet (CT), a high fat diet (HF) or a high fat diet supplemented with 10% arabinoxylan (HF-AX) for 4 weeks. (DOC) [file pone.0020944.s005.doc]

|  | HF | **HF-AX** |
| --- | --- | --- |
| *Hepatic gene expression* |  |  |
| - SREBP1c | 2.14 ± 0.25 * | 1.90 ± 0.17 * |
| - FAS | 1.34 ± 0.13 * | 1.24 ± 0.11 |
| - PPARα | 1.22 ± 0.04 * | 0.97 ± 0.06 § |
| - CPT1 | 1.11 ± 0.06 | 0.98 ± 0.06 |
| - ACO | 1.07 ± 0.04 | 0.98 ± 0.06 |
| - UCP2 | 1.15 ± 0.09 | 1.28 ± 0.09 |
| - LDL receptor | 0.90 ± 0.10 | 0.93 ± 0.08 |
| - HMGCoA reductase | 1.41 ± 0.32 | 1.58 ± 0.16 |
| - ABCG5 | 1.22 ± 0.08 | 1.03 ± 0.04 |
| - ABCG8 | 1.14 ± 0.10 | 1.14 ± 0.09 |
| - Cyp7a1 | 1.54 ± 0.26 | 1.54 ± 0.23 |
| *Muscle gene expression* |  |  |
| - PPARα | 1.29 ± 0.38 | 0.95 ± 0.11 |
| - PPARδ | 1.12 ± 0.14 | 0.92 ± 0.16 |
| - CPT1 | 1.03 ± 0.05 | 1.26 ± 0.12 |
| - ACO | 0.97 ± 0.06 | 1.07 ± 0.07 |

Values are expressed relative to controls (set at 1). * p<0.05 versus CT and ^§^p<0.05 versus HF (ANOVA). FAS, Fatty acid synthase ; CPT-1, carnitine palmitoyl transferase-1 ; ACO, AcylCoA oxydase ; UCP-2, uncoupling protein-2 ; LDL, low density lipoprotein ; PPAR , peroxisome proliferator-activated receptor ; SREBP-1c, sterol regulatory element binding protein-1c
