# Supplementary material for: Prebiotic Effects of Wheat Arabinoxylan Related to the Increase in Bifidobacteria, Roseburia and Bacteroides/Prevotella in Diet-Induced Obese Mice
Source: PLoS One. 2011 Jun 9;6(6):e20944. doi: 10.1371/journal.pone.0020944 (PMC3111466; doi:10.1371/journal.pone.0020944)
Supplement: Table S3 — Analysis of correlation between the number of bacteria in the caecal content (expressed as Log10 (bacterial cells/ total caecal content wet weight) with metabolic parameters that were significantly affected by AX treatment. (DOC) [file pone.0020944.s006.doc]

| **Host parameters** | | **Bifidobacteria** | | | ***Roseburia* spp.** | | | ***Bacteroides/Prevotella* spp.** | | |
| --- | --- | --- | --- | --- | --- | --- | --- | --- | --- | --- |
|  |  | **Pearson r** | **P value** |  | **Pearson r** | **P value** |  | **Pearson r** | **P value** |  |
| Weight | BW gain | 0,3389 | 0,1137 |  | -0,7023 | 0,0002 | ** | -0,6292 | 0,0013 | * |
|  | subcutaneous adipose tissue | 0,4725 | 0,0228 |  | -0,7348 | P<0.0001 | *** | -0,5822 | 0,0036 | * |
| Serum | Insulin resistance index | 0,4149 | 0,0549 |  | -0,6531 | 0,001 | ** | -0,4459 | 0,0375 |  |
|  | adiponectin | -0,3356 | 0,1481 |  | -0,0886 | 0,7102 |  | -0,2701 | 0,2494 |  |
|  | IL6 | -0.5960 | 0.0034 | * | 0.2519 | 0.2581 |  | 0.0782 | 0.7294 |  |
|  | MCP-1 | -0.6951 | 0.0003 | ** | 0.3399 | 0.1217 |  | 0.1275 | 0.5717 |  |
|  | cholesterol | 0,4142 | 0,0494 |  | -0,8193 | P<0.0001 | *** | -0,6803 | 0,0004 | ** |
|  | HDL-cholesterol | 0,1237 | 0,574 |  | -0,6616 | 0,0006 | ** | -0,6469 | 0,0009 | ** |
|  | LDL-cholesterol | 0,4837 | 0,0226 |  | -0,8131 | P<0.0001 | *** | -0,7564 | P<0.0001 | *** |
| Liver content | free cholesterol | 0,0252 | 0,9093 |  | -0,4874 | 0,0183 |  | -0,5249 | 0,0101 |  |
| Liver mRNA | PPARα | -0,1426 | 0,5164 |  | -0,5372 | 0,0082 | * | -0,4027 | 0,0568 |  |
| Jejunum   mRNA | ZO1 | 0,7749 | P<0.0001 | *** | -0,0222 | 0,92 |  | -0,1145 | 0,6028 |  |
|  | occludin | 0,6772 | 0,0005 | ** | -0,0468 | 0,836 |  | -0,0106 | 0,9627 |  |
| Adipocyte number/area | | -0,3711 | 0,0891 |  | 0,7269 | 0,0001 | ** | 0,578 | 0,0048 | * |
| WAT  content | SFA | -0,5070 | 0,0136 |  | 0,4238 | 0,0439 |  | 0,1379 | 0,5303 |  |
|  | rumenic acid | 0,6638 | 0,0006 | ** | -0,6084 | 0,0021 | * | -0,3933 | 0,0634 |  |
| WAT  mRNA | F4/80 | 0,1803 | 0,4220 |  | -0,5394 | 0,0096 | * | -0,6747 | 0,0006 | ** |
|  | IL-6 | 0,1945 | 0,3858 |  | -0,7126 | 0,0002 | ** | -0,5441 | 0,0089 | * |
|  | PPARγ | 0,1860 | 0,4073 |  | -0,6257 | 0,0018 | * | -0,5729 | 0,0053 | * |
|  | aP2 | -0,0675 | 0,7652 |  | -0,6632 | 0,0008 | ** | -0,6303 | 0,0017 | * |
|  | GPR-43 | 0,1313 | 0,5603 |  | -0,7064 | 0,0002 | ** | -0,6040 | 0,0029 | * |
|  | CPT-1 | 0,3358 | 0,1367 |  | -0,6707 | 0,0009 | ** | -0,6306 | 0,0022 | * |
|  | LPL | -0,0539 | 0,8165 |  | -0,7037 | 0,0004 | ** | -0,7221 | 0,0002 | ** |
|  | CD36 | 0,0812 | 0,7264 |  | -0,755 | P<0.0001 | *** | -0,6832 | 0,0006 | ** |
|  | ACO | 0,1960 | 0,3945 |  | -0,6148 | 0,003 | * | -0,5345 | 0,0126 |  |
|  | FAS | -0,5636 | 0,0078 | * | 0,2820 | 0,2156 |  | 0,2427 | 0,2892 |  |
|  | MGL | 0,0770 | 0,747 |  | -0,8451 | P<0.0001 | *** | -0,7034 | 0,0005 | ** |
| WAT activity | FAS | -0,5278 | 0,0096 | * | -0,0058 | 0,9792 |  | 0,0979 | 0,6566 |  |

Statistical significance: *p<0.01, **p<0.001 and ***p<0.0001 with the absolute value of Pearson r > 0.5

PPAR , peroxisome proliferator-activated receptor ; aP2, adipocyte fatty acid binding protein 4 ; GPR43, G protein-coupled receptor 43; LPL, lipoprotein lipase ; CD-36, cluster of differenciation 36 ; FAS, Fatty acid synthase; CPT-1, carnitine palmitoyl transferase-1 ; ACO, AcylCoA oxydase ; MCP-1, monocyte chemoattractant protein-1; MGL, monoacylglycerol lipase ; HDL-high density lipoprotein ; LDL, low density lipoprotein ; SFA, saturated fatty acids; WAT, white adipose tissue ; ZO1, zonula occludens 1
